# Prognostic value of cortically induced motor evoked activity by TMS in chronic stroke: Caveats from a revealing single clinical case

**DOI:** 10.1186/1471-2377-12-35

**Published:** 2012-06-08

**Authors:** Julià L Amengual, Antoni Valero-Cabré, Misericordia Veciana de las Heras, Nurja Rojo, Seán Froudist-Walsh, Pablo Ripollés, Nils Bodammer, Bahram Mohammadi, Jordi Montero, Carles Grau, Thomas F Münte, Antoni Rodríguez-Fornells

**Affiliations:** 1 Neurodynamic Laboratory, Departament of Psychiatry and Clinical Psychobiology Universitat de Barcelona, 08035, Barcelona, Spain; 2 Cognition and Brain Plasticity Unit. Bellvitge Research Biomedical Institute (IDIBELL), Hospitalet de Llobregat, Llobregat, Spain; 3 Université Pierre et Marie Curie, CNRS UMR 7225-INSERM UMRS 975, Groupe Centre de Recherche de l’Institut du Cerveau et la Möelle (ICM), Paris, France; 4 Laboratory for Cerebral Dynamics Plasticity & Rehabilitation, Boston University School of Medicine, Boston, USA; 5 Cognitive Neuroscience and Information Technology Research Program Open University of Catalonia (UOC), Barcelona, Spain; 6 Hospital Universitary de Bellvitge (HUB), Neurology Section, Campus Bellvitge University of Barcelon, Barcelona, Spain; 7 Dept. of Basic Psychology, University of Barcelona, 08035, Barcelona, Spain; 8 Unit of Cognitive Neurology and Aphasia, Centro de Investigaciones Médico-Sanitarias (CIMES) University of Malaga, Campus Teatinos, Málaga, 29010, Spain; 9 Department of Psychosis Studies, Institute of Psychiatry, King's Health Partners, King's College London, London, United Kingdom; 10 Center for Lifespan Psychology, Max Planck Institute for Human Development, Berlin, Germany; 11 Department of Neurology, University of Lübeck, Lübeck, Germany; 12 CNS-LAB, International Neuroscience Institute (INI), Hannover, Germany; 13 Institució Catalana de Recerca i Estudis Avançats (ICREA), Barcelona, Spain

## Abstract

**Background:**

We report the case of a chronic stroke patient (62 months after injury) showing total absence of motor activity evoked by transcranial magnetic stimulation (TMS) of spared regions of the left motor cortex, but near-to-complete recovery of motor abilities in the affected hand.

**Case presentation:**

Multimodal investigations included detailed TMS based motor mapping, motor evoked potentials (MEP), and Cortical Silent period (CSP) as well as functional magnetic resonance imaging (fMRI) of motor activity, MRI based lesion analysis and Diffusion Tensor Imaging (DTI) Tractography of corticospinal tract (CST). Anatomical analysis revealed a left hemisphere subinsular lesion interrupting the descending left CST at the level of the internal capsule. The absence of MEPs after intense TMS pulses to the ipsilesional M1, and the reversible suppression of ongoing electromyographic (EMG) activity (indexed by CSP) demonstrate a weak modulation of subcortical systems by the ipsilesional left frontal cortex, but an inability to induce efficient descending volleys from those cortical locations to right hand and forearm muscles. Functional MRI recordings under grasping and finger tapping patterns involving the affected hand showed slight signs of subcortical recruitment, as compared to the unaffected hand and hemisphere, as well as the expected cortical activations.

**Conclusions:**

The potential sources of motor voluntary activity for the affected hand in absence of MEPs are discussed. We conclude that multimodal analysis may contribute to a more accurate prognosis of stroke patients.

## Background

Motor recovery following stroke is highly variable and difficult to predict from clinical symptoms [1]. The advent of several mapping techniques such as functional magnetic resonance imaging (fMRI) and Transcranial Magnetic Stimulation (TMS) may improve the exploration of recovery mechanisms [2]. For example, TMS has been used to probe corticospinal physiology and to map primary motor cortex (M1) representations of upper limb muscles following stroke. As compared to its contralesional counterparts, spared regions within the ipsilesional M1 typically show higher stimulation thresholds, prolonged latencies [3,4], and lower motor evoked potential (MEP) amplitudes on the target muscle. Those changes are thought to emerge from tissue loss in the descending corticospinal pathway or the associated cortical and subcortical structures [5]. Interestingly, MEP changes have been found to be predictive of poor motor outcomes during the first days following a stroke [6] or even in chronic stages [7]. Nonetheless, the detailed relationship between corticospinal excitability as measured by TMS and the potential for motor recovery remains unclear. In particular, in some “paradoxical” cases TMS-evoked MEP can be completely abolished in spite of fully restored motor function [8].

To address such paradoxical situations, multimodal techniques and motor mapping approaches integrating whole brain functional and structural neuroimaging with neurostimulation techniques could prove useful. For example, they might show additional activation of contralesional cortical regions that might contribute to recovery. FMRI has been used to assess the presence and distribution of cortical activity during the use of an affected upper limb, revealing that ipsilesional premotor and supplementary motor areas (SMAs) are likely to be recruited following stroke [9]. Furthermore, patients experiencing meaningful gains in motor function with physical therapy exhibit increases in ipsilesional activity, [10] whereas in those with poor motor outcomes cortical activity remains bilateral [11]. T1-weighted structural MRI can be used to determine the structural integrity of the corticospinal tract (CST), while Diffusion Tensor Imaging (DTI) tractography allows for the visualization of 3day models of the CST and quantification of white-matter pathway integrity [12,13]. Here we report on a patient who had suffered a stroke leading to a right hemiparesis 62 months prior to the investigation. His motor function had improved to near normal levels at the time of the study as demonstrated by 3day kinematic analysis. In spite of good clinical recovery, a thorough TMS exploration showed a total absence of MEP responses after stimulation of the motor cortex of the affected hemisphere. We hypothesized that this clinical-MEP dissociation might be explained by neuroimaging-based assessment of the CST. Such an assessment might prove useful in patients in which an absence of TMS-induced MEPs prevents the functional assessment of CST function [8].

## Case presentation

**Figure 1:**
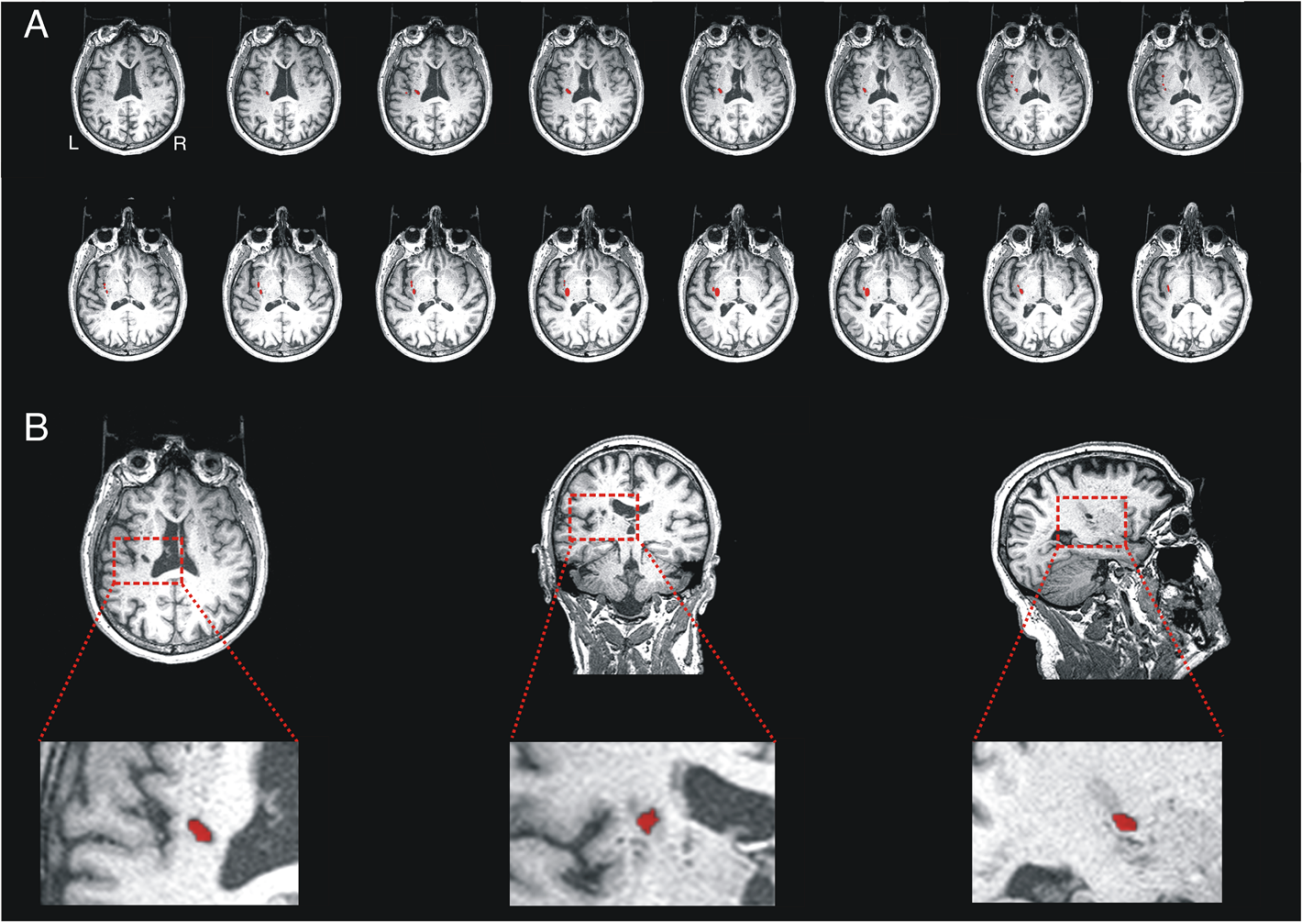
**A. Slice-by-slice reconstruction of the axial T1-weighted image of the lesion.** B. Axial, coronal and sagittal slices of the lesion, located on left hemisphere lesion at subinsular region level, including the left internal and external capsule, corona radiata and claustrum.

A right-handed 64 year-old man suffered an ischemic stroke affecting the left subinsular region and the claustrum 62 months prior to study onset (Figure 1), which resulted in a right-sided brachiofacial hemiparesis. Handedness was measured with the Edinburgh Handedness Inventory. The patient scored 10/10, indicating right-handedness. A review of the patient’s clinical record revealed a score of 3/5 for right shoulder and 2/5 for right hand strength (OMSS, Oxford Muscle Strength Scale, scored between 0 for complete paralysis, and 5 for normal strength) when he was first admitted to hospital after stroke. The clinical record also documented a mild paresis of the right lower limb (4/5). In addition, the neurologist on-duty observed a right-sided supranuclear facial paresis. Sensory function was spared and no sensory abnormalities or impairments were reported or documented in any bodily region. Deep tendon-reflexes were found to be more pronounced and brisk on the right lower limb than in the left. There was a plantar extensor reflex (Babinski sign present) only in the right lower limb. Concurrently, the patient presented with insulin-dependent diabetes and moderate elevation of liver enzymes. He was enrolled in a clinical trial evaluating motor responses. In a neurological examination prior to his participation in the evaluations performed for this study, strength in his right shoulder, right hand and right inferior were judged to be normal, with a OMSS score of 5/5. No other changes were reported. A detailed examination of the patient’s medical records did not reveal any event of epileptic seizures prior or after the stroke, nor antiepileptic medication, which could have interfered with TMS-evoked activity. At the time of the evaluation, the patient was however taking ‘Clopidogrel’ (75 mg/day), which is an oral thienopyridine class antiplatelet agent used to inhibit blood clots in coronary artery disease and cerebrovascular disease. Nonetheless, no relation between this medication and changes in cortical excitability has ever been reported. In addition, prior to the advent of the stroke the patient was taking ‘Enalapril’ (5 mg/day), an angiotensine converting enzyme inhibitor. Enalapril has never been related to changes in cortical excitability. As it was removed from the patient’s treatment after the stroke, it is very unlikely that it could have interfered TMS evoked electrophysiological recordings. Three months after the stroke, and prior to his participation in our study, the patient was enrolled in a conventional rehabilitation program for 5 months. During this period, he attended 5 days per week rehabilitation sessions at Hospital Universitari de Bellvitge. Each session lasted around 45 min. The patient’s neurological status was characterized by means of extensive motor evaluations, neuropsychological testing, fMRI during motor activity, whole brain DTI, and TMS.

### Evaluation of motor behavior

The assessment of motor function comprised clinical motor assessments and computerised tests. Clinical assessments, presented in the following order, included the Action Research Arm Test (ARAT) [14], Arm Paresis Score (APS) [15], the Box and Block Test (BBT) [16], and the Nine Hole Pegboard Test (9HPT) [17,18]. A computerised 3D movement analysis procedure (CMS 30 P, Zebris, Isny, Germany) was used to assess the spatial trajectories of tiny ultrasound markers attached to the moving body parts. Three diadochokinetic hand movements were tested with this device: alternate forearm pronation and supination, whole-hand tapping and index finger tapping [19]. Two ultrasonic markers were used for each task, and the spatial coordinates of both markers were sampled at 66 Hz, each at a spatial resolution of 0.1 mm. Continuous calculation of the three-dimensional positions of each sender was performed with commercially available software (WinData 2.19.3x, Zebris). Recording and analysis procedures were performed according to previously published methods [19]. First, the examiner demonstrated each movement. Then the subject performed four short trials, each lasting 4 to 5 seconds, with a short break of approximately 5 seconds in between. In all tests, the first trial was considered to be a practice trial, and the following three trials were recorded and used for analysis. For both affected and unaffected hands, the order of the examination was fixed, starting with finger tapping, followed by hand tapping, with forearm pronation and supination performed last. The patient was instructed to move as fast as possible.

Data analysis (Software “3DA-Version 1.2”, C. Marquardt, Munich, Germany) was performed on five series of movement cycles. Two parameters were used for each diadochokinetic task: frequency (FR), defined as the number of cycles per second, and the number of inversions of the velocity profile (NIV) per movement segment. The latter was considered as a measure of smoothness. Values of NIV close to 1 were considered optimal, whereas inversions with amplitudes less than 3% of the maximal velocity were excluded.

### Clinical motor function tests were performed once in an independent session of the computerised tests. Both sessions were performed within the same week

As an outcome measure we compared scores between the affected (right) and the unaffected (left) upper limb. Clinical motor scores used for such comparisons are presented in absolute values. In addition, the patient’s clinical motor function scores were compared to normative values from a population of healthy subjects at similar ages age to the case reported in this study [18]. Results of the computerised movement analysis are presented as mean ± standard deviation.

### Electrophysiological recordings and data analysis

Single-pulse TMS was performed using a standard 70 mm figure-of-eight coil (9 cm diameter per wing) attached to a Magstim Rapid2 Stimulator (Magstim Company, Carmathenshire, Wales UK). MEPs were recorded with surface Ag/AgCl disk electrodes in a belly-tendon montage from both left and right first dorsal interossei (FDI). This muscle was selected due to its essential involvement in skillful finger movements. Such movements were impaired in this patient as a result of his stroke. For this reason the left and right first dorsal interossei (FDI) were chosen as sites for electrophysiological recordings. In addition, both flexor carpi radiali (FCR) and both biceps brachii (BB) were examined to explore differential recovery processes for muscles proximal to the FDI. In order to record any ipsilateral activation, EMG traces from left/right FDI and left/right MBB were recorded from both sides at the same time. For each pulse, we collected EMG activity for a total of 700 ms including a 100 ms pre-stimulus window (Medelec Synergy, Oxford Instruments, Pleasantville, NY, USA). EMG activity was sampled at a 5 Khz and filtered with a band-pass of 1–1000 Hz. Data was stored and exported for off-line analysis using specialised software (Matlab ©, Mathworks, Natick, Massachusetts, USA).

An elastic lycra cap was fitted to the patient's head, on which a 10 x 10 cm grid centred on the vertex (Cz position of the international 10/20 EEG positioning system) was drawn to allow simple identification of stimulation coordinates, each of which had a 1 cm gap on all four sides to any other stimulation point. Midline points of the grid were distributed 7 cm anterior and 3 cm posterior to the vertex. From each point on the midline, 10 points separated by one cm were distributed laterally for each hemisphere. The TMS coil was placed tangential to each site, with the handle pointing backwards (in a lateral to medial and caudal to rostral position) ~45° lateral to the interhemispheric midline. Both, the damaged and the spared hemisphere were tested. For each hemisphere we determined the resting motor threshold (RMT) and the active motor threshold (AMT) for FDI and FCR. MEP latencies were calculated for FDI, FCR and BB. Intracortical inhibition was assessed by using the cortical silent period (CSP) for contralateral FDI and FCR muscles. We determined the centre of gravity (CoG) of the motor mapping representation for the FDI hotspot in the unaffected hemisphere. We recorded the length of the absolute cortical silent period (CSP) registered at the contralateral FDI for both hemispheres, and its topographical distribution.

For FDI and FCR of either arm, the motor hot spot was defined as the location where the highest MEP amplitude could be elicited. The RMT was measured as the minimum stimulus intensity inducing an MEP of at least 50 μV in 50% of 10 trials at rest at the motor hot spot [20]. The AMT was defined as the minimum stimulus intensity leading to a MEP of at least ~200 μV in 5 of 10 trials during an isometric muscle activation at about 10% of maximum voluntary contraction, measured using a pressure gauge (Baseline Evaluation Systems, WA, U S A). For the CSP study, stimuli were delivered at maximum stimulation output, while the patient performed a unilateral isometric activation of the target muscle at about 10% of maximum voluntary contraction. Fifteen consecutive motor responses were recorded at variable intervals of at least 7 to 10 seconds between pulses. The CSP duration was estimated as the interval from the TMS stimulus to the time at which the post-MEP EMG activity (high-pass filtered and squared) reached 25% of the average pre-stimulus level [21]. MEP latencies were calculated for each muscle as the interval between the pulse artefact and the first MEP deflation after stimuli delivered at the hot spot at 120% of the RMT. Five consecutive motor responses were recorded at rest, recorded at variable intervals 7 to 10 seconds. Maps were generated by plotting the peak-to-peak MEP amplitude as a function of the stimulated scalp sites. Sites were located from the grid using a latitude/longitude-based coordinate system [22]. In the original grid, locations spaced 2 cm in latitude and 2 cm in longitude were stimulated. During mapping, consecutive series of 5 stimuli spaced 7 to 10 seconds apart were delivered at each scalp site, with the muscle in a relaxed state. Pulses were delivered up to 100% of the maximal stimulator output, and no MEP was ever evoked in the affected hemisphere. Pulses were delivered to the unlesioned hemisphere at 120% of RMT. CoG was determined as the amplitude-weighted centre of the MEP amplitude map (see [23] for further details).

The lack of any observable MEP after maximal TMS stimulation of the affected hemisphere prompted us to explore potential cortical sites related to modifications of the FDI cortical silent period. Locations of the grid as considered for motor mapping were explored. Five TMS stimuli 7–10 seconds apart were delivered at each scalp site, while the patient performed a voluntary contraction of the FDI muscle at 10% of the maximal voluntary contraction. Pulses were delivered at 100% of the stimulator output for the affected hemisphere and at 120% of the RMT for the unaffected hemisphere. In these maps, a CoG was determined as the weighted center of the CSP length map throughout sites, by using similar procedures as the above described MEP amplitude mapping.

Values for cortical silent period and MEP latencies are presented as average ± standard deviation across different blocks of testing, whereas the rest of electrophysiological tests are presented as absolute threshold values. CoG coordinates for each hemisphere were presented as latitude/longitude location on the scalp.

In order to evaluate the integrity of the peripheral nerve conduction (which may have explained the lack of cortically evoked MEPs from the affected (left) right motor cortex in this patient) transcutaneous electrical stimulation was used to record the latency and amplitude of the supramaximal compound muscle action potential (CMAP) of the right and left extensor carpi ulnaris muscles. We then estimated peripheral nerve conduction time (i.e., the spinal motor neuron-to muscle latency) by recording the antidromic F-wave of the ulnar nerve and applying the following formula: (F + M-1)/2, where F and M were the shortest F- and and M-wave latencies obtained by supramaximal anodal stimulation of the ulnar nerve at the wrist level. Finally, we calculated the central motor conduction time (CMCT) for the unaffected (right) hemisphere using the formula CMCT = LC-(F + M-1)/2 [23], where LC is the latency of the onset of the MEP in right FDI muscle after magnetic stimulation.

We acquired EMG signals during unilateral finger-to-thumb pinch and grasping movements to assess in a more objective manner if the engagement of the healthy unaffected (left) hand in voluntary activity could induce coupled EMG activity in the affected (right) hand and forearm and vice-versa. We attached Ag/AgCl electrodes to the left (unaffected) and right (affected) FDI and FCR muscles in a belly-tendon montage. We first asked the patient to perform with the unaffected (left) hand a set of 10 pinch movements. Right after this, the patient performed a set of 10 grasping movements. The same procedure was used thereafter with the affected (right) hand. In order to regularly pace the motor activity of the patient and equate as much as possible such rhythms to the motor activity patterns tested in other sections of the manuscript we asked him to emulate the performance of an investigator placed in front on him at a constant pace of one movement each 3–4 seconds. The EMG recordings corresponding to 1 second of the 10 individual movement cycles for pinching and grasping were automatically time-locked at the time point in which the EMG traces showed muscle activity in the commanding hand of at least 200 μV, and once aligned in time, the 10 recordings were averaged through. The whole session was videotaped to document potential macroscopic evidence of coupled mirror motor activity

### fMRI scanning and analysis procedure

The fMRI session comprised two motor tasks using a block design. The Grasping task required a grasping movement with the right or left hand alternating with blocks of rest (4 blocks, 20 seconds per block, per active condition in a single run of approximately 6 minutes). The Tapping task required tapping movements with the index finger of the right or left hand interleaved with blocks of rest (3 active blocks, 20 seconds per block, three runs of approximately 3 minutes each).

Images were obtained with a 3 T whole-body MRI scanner (Siemens Magnetom Trio located at Clinic Hospital, Barcelona) equipped with a non-ferromagnetic response box. Conventional high-resolution structural images [magnetization-prepared, rapid-acquired gradient echoes (MPRAGE) sequence, 240 slices sagittal, TR = 2300 ms, TE = 3 ms, 1 mm thickness (isotropic voxels)] were followed by functional images sensitive to blood oxygenation level-dependent contrast (echo planar T2*-weighted gradient echo sequence, TR = 2000 ms, TE = 29 ms, slice thickness = 4 mm). Each functional run consisted of 176 sequential whole-brain volumes for the grasping task and 96 volumes for the tapping task. Each volume comprised 32 axial slices aligned to the plane intersecting the anterior and posterior commissures, 3.5 mm in-plane resolution, 4 mm thickness, no gap, positioned to cover all but the most superior region of the brain and the cerebellum.

FMRI data were analyzed using standard procedures implemented in the Statistical Parameter Mapping software (SPM2, http://www.fil.ion.ucl.ac.uk/spm). The preprocessing included slice-timing, realignment, normalization and smoothing. First, functional volumes were phase shifted in time with reference to the first slice to minimize purely acquisition-dependent signal-variations across slices. Head-movement artifacts were corrected based on an affine rigid body transformation, where the reference volume was the first image of the first run (e.g. [24]). Functional data were then averaged and the mean functional image was normalized to a standard stereotaxic space using the EPI derived MNI template (ICBM 152, Montreal Neurological Institute) provided by SPM2, after an initial 12-parameter affine transformation. The resulting normalization parameters derived for the mean image were applied to the whole functional set. Finally, functional EPI volumes were re-sampled into 2 mm voxels and then spatially smoothed with an 8 mm full-width half-maximum (FWHM) isotropic Gaussian Kernel to minimize effects of inter-subject anatomical differences.

The statistical evaluation was based on a least-square estimation using the general linear model by modelling the different conditions with a box-car regressor waveform convolved with a canonical hemodynamic response function [25]. Thus, a block-related design matrix was created including the conditions of interest (Grasping task: Right grasping, Left grasping and Rest; Tapping task: Right tapping, Left tapping and Rest). Eight regions of interest (ROIs) were defined on the anatomical images of the patient in order to quantify the numbers of voxels that were activated in response to the motor tasks. The following ROIs for each hemisphere were generated using WFU pickatlas toolbox [26] for SPM: (1) Primary motor cortex (M1); (2) Supplementary Motor Area (SMA) and Premotor Cortex (PMC); (3) Anterior Cingulate cortex; (4) Cerebellum; (5) Superior Parietal Cortex; (6) Inferior Parietal Cortex; (7) Pons and (8) Midbrain.

### Diffusion tensor imaging and analysis procedure

DTI data were collected in the same scanner by an eight-channel phased array head coil with parallel imaging (GRAPPA) and an acceleration factor of 2. Diffusion weighting was conducted using the standard twice-refocused spin echo sequence. Images were measured using the following parameters: 2-mm-thick slices; no gap; TR = 9100 ms; TE = 92 ms; 128 x 128 acquisition matrix; field of view, 240 x 240 mm; 64 axial slices. To obtain diffusion tensors, diffusion was measured along 20 non-collinear directions, chosen according to the standard Siemens DTI acquisition scheme using a single b value of 1000 s/mm^2^. Two runs of the DTI data were recorded. Data was processed as follows. The images were first skull-stripped using FSL's BET [27]. The two runs of diffusion data were first concatenated, and then Eddy-current- and motion-corrected using FSL's FDT (http://www.fmrib.ox.ac.uk/fsl/fdt). The b-vectors were then rotated in order to take into account the corrections made at the previous stage.

The diffusion tensors were then reconstructed using Diffusion Toolkit's least-square estimation algorithm for each voxel (Ruopeng Wang, Van J. Wedeen, TrackVis.org, Martinos Center for Biomedical Imaging, Massachusetts General Hospital). The whole brain tractography used an interpolated-streamline algorithm with an angular threshold of 35 degrees and an FA threshold of 0.2. The tensor was spectrally decomposed in order to obtain its eigenvalues and eigenvectors. The fiber direction is assumed to correspond to the principal eigenvector (the eigenvector with the largest eigenvalue). This vector was colour coded (green for anterior-posterior, blue for superior-inferior and red for left-right) to generate a colour FA map. An FA map was also generated from these eigenvalues using Diffusion Toolkit. The motor fibers were selected using three ROIs. The first two ROIs were placed in the cerebral peduncle and the posterior limb of the internal capsule, using the color-coded FA map to guide the placement. The third ROI encompassed the pre-central cortex and its underlying white matter, and was drawn on the diffusion-weighted image (DWI), with the patient's anatomical T1-weighted image (attained with the MPRAGE sequence) used as a reference. Any artifactual fibers were removed using an exclusion ROI.

## Results

### Lesion description

**Figure 2:**
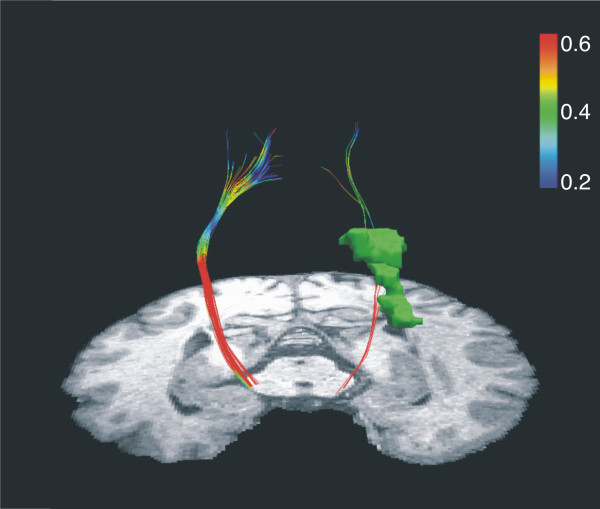
**Plot of the reconstruction of the pyramidal tract for left (Lesioned) and right (Unlesioned) hemispheres from different angles of view.** The lesion (green coloured) it is shown on the left hemisphere. A clear sparsity of reconstructed streamlines in the ipsilesional hemisphere compared to the contralesional side has been observed. Warm colours represent higher fractional anisotropy; cool colours represent lower fractional anisotropy. Note the drop in FA as the left corticospinal tract passes by the posterior part of the lesion.

The T1 MRI sequence (Figure 1) strongly suggested that the internal and external capsule were both affected, as well as the claustrum. The volume of the affected region was estimated by counting the number of voxels that appeared to have non-normal intensity. This gave an estimated lesion volume of ~1313 mm^3^ (See Figure 2, green area).

### Motor assessment results

Data from various motor tasks revealed that the patient presented a mild affected level of performance for fine and gross movement when tasks were performed with the affected right hand. Clinical motor scores showed small differences between hemispheres for the 9HTP test, which were within the normal range [18]. The rest of non-computerised motor assessments did not show any differences between hands (see Table 1 for further details).

**Table 1:** Scores for the non-computerised motor tasks

**Task**	**Unaffected side (Left)**	**Normal Values (Left)**	**Affected side (Right)**	**Normal Values (Right)**
B & B	59	68.4 (7.1)	61	67.4 (7.8)
9HTP	29	22.29 (3.7)	20	21.2 (3.29)
APS	7	7	7	7
ARAT	18, 12, 18, 9	18, 12, 18,9	18, 12, 18, 9	18, 12, 18, 9

Each score measured the performance for fine and gross movement with the affected and the unaffected hand. Data have been compared with expected values for healthy subjects within the same age range as the patient. Normal values are expressed as mean (std). Scores obtained from the subject are absolute values. (B & B: Block and Block Test; 9 HPT: Nine Hole Pegboard Test; APS: Arm Paresis Score; ARAT: Arm Research Arm Test (Pinch, Grasp, Grip, Gross Movement)).

**Figure 3:**
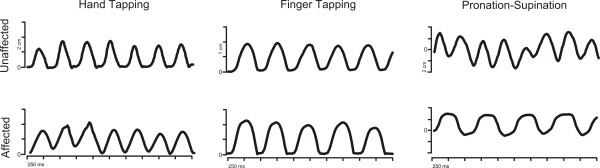
**Mediolateral components (Finger and Hand Tapping) and anteroposteror (Pronation-Supination) of the movement of one marker during the task performance**, **for the affected and the unaffected hand.** Differences of the quality of the performance between both hands are observable.

Results of the computerised movement analysis are summarised in Table 2. We found differences in frequency in the forearm pronation and supination task and NIV during hand tapping task between the affected (right) and unaffected (left) hands (See Figure 3). We did not find differences between the two sides on any other measure.

**Table 2:** Frequency and NIV of different diadochokinetic movements measured using a 3D ultrasound movement analysing device

**Task**	**Pronation/Supination (PS)**	**Hand Tapping (HT)**	**Finger Tapping (FT)**
Frequency Affected (FREQ)	1.5 (0.4)	3 (0.3)	2.4 (0.1)
Frequency Unaffected (FREQ)	3.4 (0.5)	2.9 (0.2)	3.6 (0.3)
Δ FREQ	1.9	−0.1	1.2
NIV Affected	1.2 (0.2)	1.6 (0.2)	1.1 (0.02)
NIV Unaffected	1.2 (0.3)	1 (0)	1 (0)
Δ NIV	0	0.6	0.1

Absolute differences (Δ FREQ, Δ NIV) in both parameters for each task between the affected (Right) and the unaffected (Left) hand are shown. Values are showed as mean (std). Slight differences for FREQ in HT and for the NIV in FT and PS indicate the good performance of the motor tasks with the affected hand compared with the unaffected.

### Electrophysiological findings

**Figure 4:**
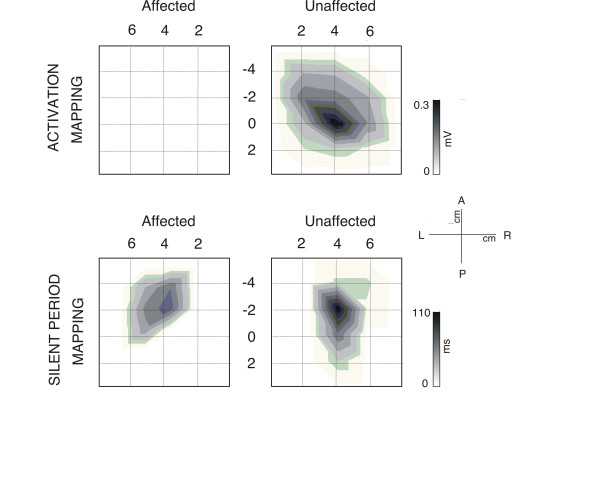
**Topographic activation and inhibitory activity maps from the unaffected and affected sides for this patient.** (Top) Maps are scaled from zero to the maximum MEP activation on each side. MEPs were only obtained after stimulation in locations within the unaffected hemisphere. (Bottom). Topographic distribution of the Cortical Silent Period (CSP) duration through the affected (left) and unaffected (right) scalp. In both hemispheres, the distribution is predominantly parallel to the anteroposterior midline. Maps are scaled from zero to the maximum duration of each side. The maximum duration on the unaffected hemisphere is longer than on the affected side.

**Figure 5:**
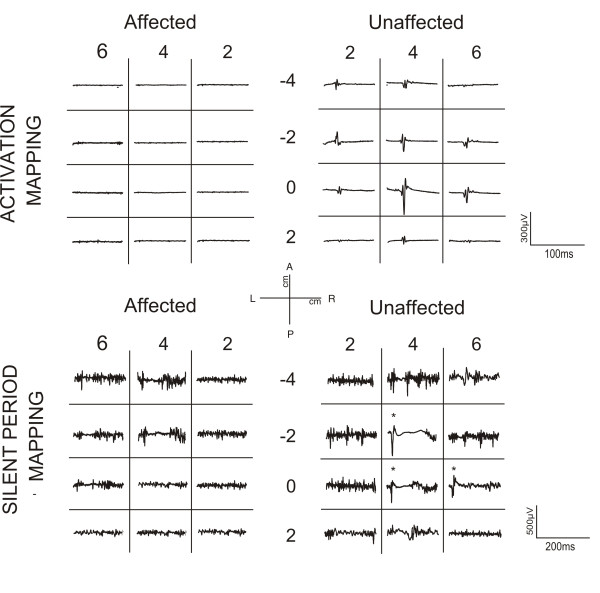
**Mapping of the activation and length of silent period of contralateral FDI muscle for affected and unaffected hemisphere.** (Top**)** In each square, the average of the EMG activity after stimulation in each location on the scalp has been plotted. Note the absence of MEP in any location of contralateral FDI after stimulation of ipsilesional side. (Bottom). Mapping of the EMG activity of contralateral FDI muscle for affected and unaffected hemisphere during voluntary contraction of the muscle. In each cell, the average of EMG activity after stimulation in each location on the scalp has been plotted. In this case, cortical silent period (CSP) on EMG in both FDI contralateral muscles after TMS pulse in several locations of the scalp is observable. However, MEP previous to the CSP only occurs reliably in the ipsilesional FDI after stimulation (starred). In all maps, the vertex is located in the origin of coordinates.

**Figure 6:**
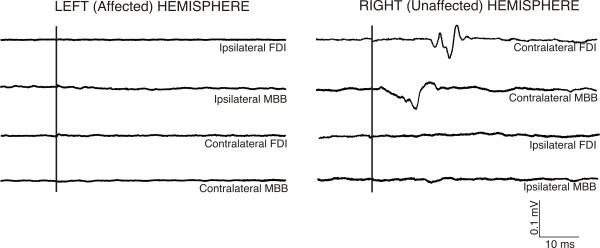
**Averages of EMG traces of muscular activity recorded from ipsilateral and contralateral FDI and MBB after TMS elicited on left hemisphere (affected) and right hemisphere (unaffected).** After pulses on right hemisphere, MEPs on contralateral FDI and MBB mucles were obtained, whereas ipsilateral muscles remained inactive. After stimulation on the left hemisphere, no MEPs were observed in ipsilateral and contralateral muscles.

In the intact right hemisphere RMT was 77% (90%) of the maximal stimulator output for the FDI (FCR). AMT was determined at 46% (65%). The MEPs displayed average latencies at the hotspot of the intact hemisphere of 21.5 ± 1.4 ms for FDI, 17.0 ± 1.6 ms for FCR and 15.3 ± 2.1 ms for MBB. The silent period duration at the hotspot was 103 ± 30 ms for the FDI, and 107 ± 27 ms for the FCR. Latitude/Longitude coordinates of the CoG were −0.1/2.4 (relative to vertex, Figure 4) for FDI motor mapping. In the lesioned (left) hemisphere no MEP could be elicited for any of the three tested muscles at the maximal output of the stimulator. In contrast, a silent period with pulses at levels of 90-100% of the maximal stimulation output could be determined in FDI and FCR, although without any signs of a MEP prior to the onset of the EMG silent period (Figure 5). Since the motor hotspot could not be clearly localised on the basis of individual MEPs at rest or under muscular activation, we calculated the maximum duration of the silent period across grid locations, which appeared shorter than that of the intact hemisphere (FDI: 88 ± 15 ms, and FCR: 73 ± 21 ms). No ipsilateral MEP activity was observed in left/right FDI or left/right MBB muscles*.* (see Figure 6).

The CSP was recorded from the scalp sites defined by the grid to obtain a topographic distribution of the intracortical inhibitory circuitry activated by the TMS pulse. Latitude/Longitude coordinates of the CoG for the CSP distribution were −2.8/4.3 for the unaffected and −2.5/3.8 for the affected hemisphere and thus very similar. The longest CSP durations were found over M1 (see Figure 4 and 5).

**Figure 7:**
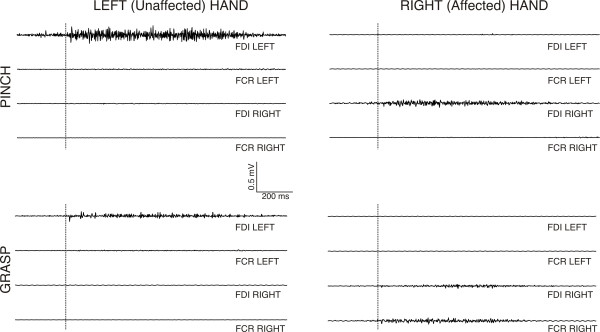
**Averaged EMG activity of left/right FDI and FCR muscles during unilateral pinch and grasp movements performed with the left and right hand.** We can see activation of the left FDI muscle during left pinch and grasp movement. During right pinch movement, right FDI muscle was activated. Right FDI and FCR muscles were activated during right grasp movements. No mirror muscle activation was observed during the performance of movements.

A detailed examination of such recordings reveals that the activation of the unaffected (left) hand during both grasping and pinching evoked EMG activity in the FDI muscle of left, but not right hand (see Figure 7). Grasping and pinching of the right hand evoked EMG activity in the right FDI muscle. In addition, right FCR muscle eventually showed a spared activation during grasp movements. Left FCR and FDI muscles remained inactive during right grasping and pinching of the right hand. We thus conclude that this patient does not show any signs of left (unaffected) to right (affected) or right to left upper limb coupled “mirror” EMG activity during grasping and pinching, which could be suggestive of the control of the affected (right) hand by the unaffected (right hemisphere).

Peripheral conduction time was calculated as (F + M-1)/2, where F and M were the shortest F- and M-wave latencies obtained by supramaximal transcutaneous anodal stimulation of the ulnar nerve. Latency of the M-wave on the left and right ulnar nerve was 2.40 and 2.45 ms. Latency of the F-wave on the left (affected corticospinal tract) and right (unaffected corticospinal tract) ulnar nerve reached values of 26.4 and 27.4 ms respectively. Peripheral conduction time for the left and right ulnar nerves was 13.0 and 14.4 ms. The shortest MEP latency obtained from the left FDI muscle was 20.1 ms. With these data in hand, we calculated the tract central motor conduction time (CMCT) [23] of the unaffected cortico-spinal tract using the formula CMCT = LC-(F + M-1)/2, where LC is the latency of the onset of the MEP following single pulse magnetic stimulation, which reached a value of 6.3 ms. The CMCT for the affected corticospinal tract could not be calculated given the inability to evoke MEP activity on affected (right) upper limb from the injured (left) hemisphere.

### fMRI recordings

**Figure 8:**
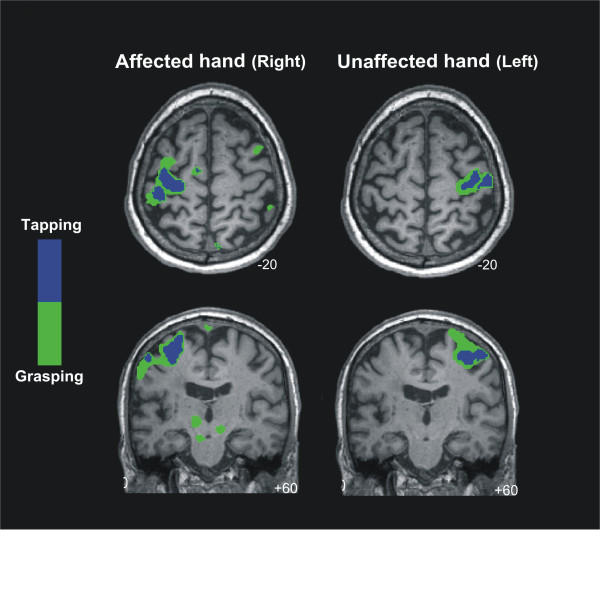
**BOLD signal changes for finger tapping and grasping movements superimposed on the individual structural MRI image in standard stereotactic space.** Green and blue colours represent the activation area of the brain for grasping and tapping movements respectively. Notice the large overlap in the contralateral sensorimotor and premotor regions (t-score overlays after multiple comparisons correction at the whole-brain level, P < 0.05).

Whole-brain analyses revealed a widespread activation of the contralateral primary sensorimotor-premotor network during grasping and tapping tasks for both sides (Figure 8, Table 3). Both tasks recruited very similar brain regions, involving cortical motor areas such as M1, SMA, pre-motor regions, and the inferior parietal cortex with the clusters of activation being smaller for the tapping task.

**Table 3:** MNI coordinates and T value for the peak location in a particular anatomical cluster

**Stereotactic coordinates**												
AFFECTED HAND			Right Grasping vs Rest					Right Tapping vs. Rest				
Brain Region	Hemisphere	BA	n. voxels	x	y	z	Tvalue	n.voxels	x	y	z	T Value
M1	Contralateral	4	322	−28	−28	68	13.68	193	−24	−28	70	10.36
SMA/PMC	Contralateral	6	277	−32	−22	68	12.03	208	−6	−10	74	7.99
Cerebellum	Contralateral		41	−20	−34	−26	7.11					
Inf. Parietal Lobe	Contralateral	39	47	−46	−36	56	8.57					
Pons	Contralateral		62	−8	−22	−24	6.79					
Midbrain	Contralateral		271	−12	−20	−6	7.53					
SMA/PMC	Ipsilateral	6	53	56	6	44	10.7					
Cerebellum	Ipsilateral		52	40	−68	−24	6.56					
Inf. Parietal Lobe	Ipsilateral	40	26	50	−42	58	7.74					
Pons	Ipsilateral		95	12	−30	−28	6.37					
Midbrain	Ipsilateral		260	−12	−20	−6	7.53					
UNFFECTED HAND		Left Grasping vs. Rest	Left Tapping vs. Rest									
Brain Region	Hemisphere	BA	n. voxels	x	y	z	Tvalue	n.voxels	x	y	z	T Value
M1	Contralateral	4	210	36	−20	54	13.71	149	40	−16	54	9.47
SMA/PMC	Contralateral	6	168	40	−18	62	12.06	173	28	−6	70	10.16
Ant. cingulate	Contralateral	24	20	4	−4	50	5.69	58	2	−6	50	9.36
SMA/PMC	Ipsilateral	6	24	−46	−6	40	7.03	91	−2	−8	54	7.44

P < 0.05; 20 voxels minimum spatial extent corrected for multiple comparisons at the whole-brain level by using a FWE rate. Also reported is the P value for the peak of activation at cluster level corrected for multiple comparisons and the number of voxels in each cluster (n. voxels).

*BA*: Brodman´s area; *M1*, primary motor cortex; *SMA*, supplementary motor area; *PMC*, premotor cortex; Ant, anterior; Sup, superior; Inf, inferior

### DTI-tractography

**Figure 9:**
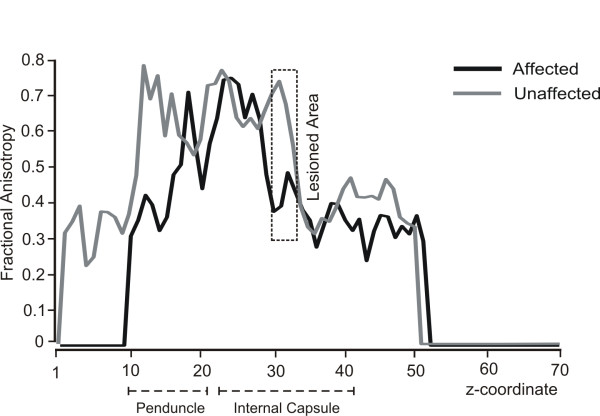
**Slice-by-slice FA values of the ipsilesional (black) and contralesional (gray) pyramidal tracts.** FA values of slices from the lesioned area on the affected hemisphere are enclosed in a dashed square. Note the differential FA values between lesioned and unlesioned track in peduncle and internal capsule regions.

Reconstruction of the pyramidal tract (Figure 2) revealed a sparsity of streamlines in the lesioned hemisphere (5 vs. 45 in the intact hemisphere) as well as a lower mean FA value (0.453 vs. 0.538). Slice-by-Slice FA values curves of the affected and the unaffected pyramidal tract are shown in Figure 9. FA values in the lesioned hemisphere tended to be lower for the cerebral peduncule region (0.37 vs. 0.61) as well as for the internal capsule (0.41 vs. 0.65). Moreover, we calculated the regional left/right cortico-spinal FA asymmetry index at the internal capsule and the cerebral peduncle regions using the following formula (FA _unaff_ – FA _aff_)/(FA _unaff_ + FA _aff_). The FA asymmetry index for the cortico-spinal tract was 0.22 at the internal capsule and 0.31 at the cerebral peduncle.

## Conclusion

We report the case of a chronic stroke patient with nearly complete recovery of motor function that showed no hand or forearm MEP activity in response to high intensity single pulse TMS stimulation of frontal areas of the injured hemisphere. We used different neuroimaging techniques (fMRI, DTI, TMS) and several methods to evaluate the motor performance, with the aim of fully describing this case and thus explain the seemingly contradictory clinical/TMS results. The most relevant results were (1) the absence of any MEP activity in contralateral FDI, FCR and BB after stimulation on the left (affected) hemisphere, (2) a mild paresis of the affected (right) fine motor performance measured with computerised and non-computerised movement tests and (3) a FA asymmetry of the cortico-spinal tract below .25 in the internal capsule. Prior studies had suggested that the appearance of MEP activity in the affected hand a few days after a stroke is associated with good prognosis [28]. Yet, in the present case, it was not possible to determine any resting (or actively facilitated) MEP activity evoked by TMS to the lesioned hemisphere, while TMS to the intact hemisphere revealed normal thresholds. The only sign of corticospinally conveyed modulatory activity observable in the affected limb came from recordings of cortical silent periods (CSP, see Figure 5). This “paradoxical” dissociation of excellently recovered motor functions and a lack of MEP responses strongly suggests that missing MEPs might not necessarily imply a poor prognosis for motor recovery. Furthermore, the dissociation raises the possibility that motor activity is generated by a plastically altered motor system involving alternative pathways. Our patient had a subcortical lesion mainly affecting the ventral portion of the internal capsule, which hosts the descending CST [29]. As reported by [30], damage in this subcortical region alone or in combination with other lesions is often associated with poor isolated hand motor function compared to lesions of the cortex, which is in contrast with the near-to-complete functional recovery in the present case.

The deterministic DTI analysis of the CST demonstrated a “blockade” at the level of the internal capsule on the affected side, revealed by a decrease in FA and number of reconstructed streamlines (See Figure 9). Moreover, the left/right cortico-spinal FA asymmetry index (calculated as FA_unaff_ – FA_aff_/FA_unaff_ + FA_aff_) at the cerebral peduncle region was above 0.25. Stinear and colleagues ([8]) reported that in stroke patients an absence of MEPs in distal muscles and a high FA asymmetry index (>0.25) in regions of CST is predictive of poor prognosis for motor recovery. Surprisingly, data from motor and behavioural tests in the present patient challenge such notion and supports an opposing view. Fine motor control of the affected hand, measured by the number of inversions of velocity per cycle (NIV) during diadocokinetic movement tasks, was close-to-normal in two of the three tested movements. However, results from the frequency tests reveal the right hand to be mildly affected.

Interestingly, differences in the FA between both corticospinal tracts were also found at locations distal and slightly proximal from the damaged area, probably caused by anterograde or retrograde axonal degeneration. However, cortical motor areas were preserved and presumably not affected by the stroke. This finding is in contrast to previous work of Klöpfel and colleagues [31] who have shown a strong correlation between the degree of white matter coherence in subcortical regions with the motor thresholds assessed by magnetic stimulation. However, recently, Hübers and colleagues [32] failed to find this correlation between TMS and DTI in a group of healthy subjects. Nonetheless, one might suspect that tractography overstates the degree of damage to CST in the present case, and indeed further studies are needed to clarify the correspondence between the integrity of the CST using TMS and DTI measures (see [33]).

Importantly, even though MEPs could not be evoked from the lesioned hemisphere, cortical regions were able to modulate motor activity in the present case, as suggested by the presence of a TMS induced silent period, which was shorter than in the spared hemisphere. Previous work has generally found that subcortical lesions result in longer cortical silent periods (CSP) compared to the spared hemisphere [34] and that longer silent periods are also correlated to worse prognosis [30]. In addition, in the affected hemisphere, we evoked a cortical silent period in absence of a MEP. It could be argued that the stimulator was not powerful enough to elicit an MEP from the lesioned hemisphere. Although we cannot rule out this hypothesis, we consider it unlikely as at least the AMT of the spared hemisphere was well below the maximal output of the stimulator (46%). Another, in our view similarly unlikely, explanation would be that the patient’s lesion blocked descending corticospinal fibers implicated in motor execution but not modulatory inhibitory activity which might be conveyed by spared descending systems.

It is important to bear in mind for the interpretation of the present case that TMS induces trans-synaptic effects, eliciting discharges in corticospinal output neurons [35]. These postsynaptic potentials travel along the CST finally reaching the target muscle and inducing a motor response. In contrast, when a voluntary motor activity is performed, sets of excitatory and inhibitory cortico-subcortical loops are involved in the preparation and execution of movement, and the mechanisms underlying the execution of this activity are far more complex than those triggered and tested by a single TMS pulse. Indeed, several structures besides M1, premotor and supplementary motor regions are involved in granting motor activity such as the striatum, thalamus, globus pallidus, substantia nigra and subthalamic nuclei [36]. Another discrepancy in the present case concerns the contrast between the TMS and the fMRI results. BOLD activity associated with finger tapping and grasping performed with the affected upper limb was located within the cortical ipsilesional sites. Interestingly, we also observed significant activity in contralesional precentral regions during the grasping task performed by the affected hand, as well as subcortical activation within the pons, the midbrain and the cerebellum. As has been documented previously, effective recovery in chronic stroke patients tends to evolve from an initial excessive activation of the contralesional hemisphere to a more ipslesional-lateralised pattern in the chronic stage. Activation in the contralesional sides is often observed in chronic stages and may be associated with the final recovery pattern [2,9]. In any case, the recovery seems to depend on the individual patients’ ability to recruit residual portions of the bilateral motor network [37].

Considering the fMRI-TMS pattern of results in the present patient, a possible explanation would be that due to plasticity effects related to this patient’s stroke recovery, voluntary motor control is shared between ipsilesional and contralesional regions [38-42]. Full voluntary motor control from the contralesional side and control of the affected CST either via crossing transcallosal fibers or the contralesional CST does not seem possible, as this patient did not show mirror movements when TMS was applied to the healthy hemisphere.

Importantly, intracortical excitability in the injured hemisphere and transcallosal connections might undergo plastic changes within 40–80 days of stroke. It has been demonstrated in animal studies that the brain stem, reticular nucleus and red nucleus are involved in voluntary motor engagement [43,44]. It is thus possible that such motor-related subcortical structures could modulate or influence voluntary (i.e. non-TMS-induced) activity in this and other cases. It is not easy to visualise the activation of the alternative motor pathways involved in motor control, for example rubro- and reticulospinal pathways with standard fMRI methods.

In sum, we propose that brain reorganization and compensatory processes after stroke in this patient might have elicited the orchestration of a more complex cortical and subcortical network for voluntary motor control in the injured hemisphere via the recruitment of silent but already existing synapses or even to the creation of new synaptic connections [45], although this issue cannot be directly supported by the current data.

Nonetheless, this idea is in agreement with existing views about how effective recovery could be achieved in stroke patients. Stroke patients might recruit an extended network comprising premotor and sensorimotor structures normally reserved for the performance of complex movements for even the simplest of gestures [46,47]. This is likely to have indirectly increased the threshold of the excitatory interneurons and corticospinal neurons in the affected hemisphere needed to induce MEP activity after single pulse TMS stimulation. This however did not affect BOLD-motor activity in the injured hemisphere. This is indeed not strange, as BOLD activity indirectly reflects local field potential activity that is thought to represent the averaged synaptic input to the dendritic tree rather than its spiking output [48]. The interpretation of BOLD activation is generally ambiguous. For example, possible differences in the BOLD signal might be caused by increased presynaptic inhibition instead of excitation [37,49]. TMS activity is instead directly producing M1 monosynaptic corticospinal commands, resulting in activation of α-motoneurons. Interestingly, the present reorganization did not affect the cortical silent period in the lesioned hemisphere, which has a cortical origin, produced by inhibitory interneneurons and reflects stimulus-induced transient inhibition of tonic muscle activity [50-52].

In conclusion, our results suggest a relationship between the motor thresholds assessed by magnetic stimulation and white matter structure in terms of FA and number of fibres. Nonetheless, more attempts to validate the use of DTI parameters to predict conductivity need to be performed. The current case also suggests that the absence of observable MEP after TMS stimulation cannot be considered by itself as predictor for motor recovery. To improve the prognostic power, TMS may be supplemented and contrasted with information from different neuroimaging techniques.

## Consent

Written informed consent was obtained from the patient for publication of this case report and any accompanying images. A copy of the written consent is available for review by the Editor-in-Chief of this journal.

## Competing interests

The authors declare that they have no competing interests.

## Author’s contribution

JA collected and analysed the TMS and computational movement analyser data, and he wrote of the present manuscript. AVC was present on the acquisition of TMS data, he designed the TMS protocol and he reviewed critically the final draft of the present manuscript. MVH assisted during the TMS acquisition and gave intellectual support on the analysis and interpretation of electrophysiological data. NR passed the non computerized movement tests to the patient, as well as gave a critical interpretation of their results. Also, she acquired and analysed the fMRI data. SFW analyzed the DTI data, and he did a critical interpretation to the DTI findings. PR helped on the analysis of the fMRI data, reviewed the present manuscript and assisted during the acquisition of the data. NB assisted the analysis of the DTI data, giving a very useful critical view to the DTI findings. BM participated on the design of the TMS protocol, and he reviewed the manuscript. JM assisted during the TMS acquisition and gave intellectual support on the analysis and interpretation of electrophysiological data. CG reviewed critically the final draft of the manuscript. TFM and ARF conceived of the study, and participated in its design and coordination and helped to draft the manuscript. All authors read and approved the final manuscript.

## Pre-publication history

The pre-publication history for this paper can be accessed here:

http://www.biomedcentral.com/1471-2377/12/35/prepub
